# Impact of Supercritical CO_2_ Treatment on Lupin Flour and Lupin Protein Isolates

**DOI:** 10.3390/foods14040675

**Published:** 2025-02-17

**Authors:** Rubén Domínguez-Valencia, Roberto Bermúdez, Mirian Pateiro, Laura Purriños, Jose Benedito, José M. Lorenzo

**Affiliations:** 1 Centro Tecnológico de la Carne de Galicia, Avda. Galicia N° 4, Parque Tecnolóxico de Galicia, 32900 San Cibrao das Viñas, Ourense, Spain; robertobermudez@ceteca.net (R.B.); mirianpateiro@ceteca.net (M.P.); laurapurrinos@ceteca.net (L.P.); jmlorenzo@ceteca.net (J.M.L.); 2 Grupo ASPA (Anàlisi I Simulació de Processos Agroalimentaris), Instituto de Ingeniería de Alimentos, Food-UPV, Universitat Politècnica de València, Camí de Vera s/n, E46022 Valencia, Spain; jjbenedi@tal.upv.es; 3 Área de Tecnoloxía dos Alimentos, Facultade de Ciencias, Universidade de Vigo, 32004 Ourense, Spain

**Keywords:** *Lupinus luteus*, supercritical CO_2_ extraction, oil reduction, chemical composition, amino acids, vegetable protein

## Abstract

Global population growth is putting pressure on the food supply, necessitating the exploration of new, alternative, and sustainable protein sources. Lupin, an underutilized legume in human nutrition, has the potential to play a significant role in addressing this challenge. However, its incorporation into the human diet requires thorough investigation, including exploring and optimizing functionalization processes to maximize its potential. This study aimed to optimize the parameters (pressure, time, and CO_2_ flow) for extracting anti-technological factors (ATFs) from lupin using supercritical CO_2_ (SC-CO_2_) and to evaluate the effects of this extraction on both the flour and the protein isolate derived from it. Optimization revealed that the optimal SC-CO_2_ conditions were a CO_2_ flow rate of 4 kg/h at 400 bar for 93 min. Under these conditions, significant changes were observed in the flour composition, including a reduction in oil, polyphenols, and moisture content, along with an increase in ash content. Improved color parameters were also noted. These variations were attributed to the removal of oil and phenolic compounds during processing. Furthermore, this research demonstrated that SC-CO_2_ treatment improved lupin protein isolate (LPI) purity (93.81 ± 0.31% vs. 87.42 ± 0.48%), significantly reduced oil content (8.31 ± 0.09% vs. 14.31 ± 0.32%), and enhanced color parameters. The SC-CO_2_ procedure also resulted in a higher protein extraction yield (56.95 ± 0.45% vs. 53.29 ± 2.37%). However, the total extraction yield (g LPI/100 g of flour) was not affected by SC-CO_2_ treatment, remaining at 24.30 ± 0.97% for the control sample and 24.21 ± 0.26% for the treated sample. The extracted oil (2.71 ± 0.11 g/100 g of flour), a co-product of the SC-CO_2_ step, exhibited a fatty acid profile characterized by high levels of unsaturated fatty acids (62.8 ± 0.74 g/100 g oil), oleic acid (27.76 ± 0.77 g/100 g oil), linoleic acid (25.98 ± 0.73 g/100 g oil), and α-linolenic acid (5.32 ± 0.16 g/100 g oil), as well as a balanced ratio of essential fatty acids (n-6/n-3 = 4.89). The treatment had minimal to no effect on amino acid content or chemical score, and the protein was characterized by high amounts of essential amino acids (334 ± 3.12 and 328 ± 1.05 mg/g protein in LPI-control and LPI-SF, respectively). These findings demonstrate that both the LPI and the oil extracted using SC-CO_2_ possess high nutritional quality and are suitable for human food applications.

## 1. Introduction

The increasing global population is a significant concern, as current food production capacities struggle to meet the growing demand for protein. Projections indicate a global population of 9.7 billion by 2050 and 10.4 billion by 2100 [1], further intensifying the demand for food and resources. This rapid population growth will likely lead to protein deficiencies [2,3]. Alternative protein sources have emerged as a promising and sustainable solution to this challenge [4,5]. Lupin is a particularly attractive option due to its high protein and dietary fiber content, coupled with low starch levels [6,7,8]. Lupin protein isolates (LPIs) can be utilized in the development of novel food ingredients with desirable sensory and functional properties, thereby enhancing the nutritional value of various foods [6,8]. Consequently, optimizing protein extraction and purification procedures is crucial for obtaining high-quality protein isolates [9,10].

However, several anti-technological factors (ATFs), including oil, polyphenols, and fiber, can negatively impact protein purity and extraction yields [5,9]. Any compounds that adversely affect the techno-functional properties, stability, or purity of protein isolates are considered ATFs and should be removed. Oil is generally considered the primary ATF in lupin seeds, as it is undesirable in the final product. Its presence leads to lipid–protein interactions, reduces purity, and can contribute to off-flavors [11]. Furthermore, the high unsaturated oil content in lupin can promote protein degradation through oxidation during storage [12,13]. Consequently, a defatting step is typically implemented prior to further processing in most studies and industrial processes for obtaining protein isolates, aiming to enhance protein extraction and isolate purity [6,14]. Similarly, phenolic compounds and lipid-soluble pigments, such as carotenoids, are also recognized as ATFs due to their negative impact on protein extraction yield, LPI purity, and color [9]. Phenolics can also bind to proteins, reducing their digestibility [5,9]. Therefore, minimizing phenolic compound levels is desirable from both nutritional and functional perspectives [15].

During LPI production, alkaline solubilization of full-fat lupin can increase the oil content of the resulting LPI, primarily due to the oil becoming incorporated into the protein matrix and remaining present in the isolated proteins [10,16]. This observation aligns with findings from other studies, which have shown that not only oil, but also other lipophilic compounds, are concentrated during LPI extraction [17]. Therefore, removing these ATFs is essential to prevent interference with protein extraction and purification [14,18]. Conventional defatting procedures for legume flours often involve the use of organic solvents, such as hexane, prior to LPI extraction [12,13,14,15,19]. However, the use of certain solvents, coupled with the moderate-to-high temperatures required for their removal from the final product, can promote protein denaturation, leading to decreased protein solubility and extraction yields [10,20]. Furthermore, solvent use presents several other significant limitations: it can leave undesirable off-flavors in the defatted meals [21], contributes to environmental pollution, poses toxicity risks to humans, is expensive, and solvent-based defatting procedures are time-consuming [11,22].

To address the limitations of solvent-based extractions, supercritical carbon dioxide (SC-CO_2_) extraction has emerged as a promising alternative due to its innovative and resource-efficient approach to defatting [11]. While the initial installation costs can be relatively high, the operating costs are generally lower [22]. The low operating temperatures also help preserve protein structure, characteristics, and functionality. SC-CO_2_ is recognized as an environmentally friendly and selective extraction technique that minimizes thermal degradation [11,21,23]. Furthermore, this technique offers improved extraction kinetics with low energy consumption, is reproducible and easily scalable, results in a solvent-free product [7,9], and requires minimal post-extraction processing [11].

In a previous study, our research group developed a simple, rapid, and effective procedure for obtaining high-purity lupin protein isolate using full-fat lupin flour [17]. We also investigated the application of SC-CO_2_ directly to extracted LPI, demonstrating significant improvements in LPI characteristics [24]. However, the application of SC-CO_2_ as a flour defatting step, which could have substantial positive effects on both the protein extraction process and the quality (purity) of the resulting isolate, was not explored. As mentioned, using SC-CO_2_ to defat flour offers several key advantages compared to traditional organic solvent methods, motivating the present study. Therefore, understanding the impact of SC-CO_2_ extraction on both lupin flour and LPI composition is crucial for promoting LPI utilization in the food industry. While other studies have investigated the effects of supercritical CO_2_ defatting on other legume flours, the application of SC-CO_2_ to extract ATFs of *L. luteus* flour remains largely unexplored. The unique characteristics of *L. luteus*, such as its lower lipid and higher protein content compared to other lupin varieties, necessitate the optimization of the SC-CO_2_ process and extraction conditions. This study hypothesizes that treating *L. luteus* flour with SC-CO_2_ will enhance the quality of the extracted protein isolates. In addition, the extracted oil, rich in phytonutrients, will be characterized to assess its potential applications in the food industry, allowing for a complete valorization of *L. luteus*.

Therefore, the primary objective of this study was to optimize the extraction ATFs process of lupin flour to improve the quality of the resulting lupin protein isolate. To achieve this, key supercritical extraction conditions—including time, pressure, and CO_2_ flow—were optimized using a Box–Behnken experimental design and response surface methodology, a powerful statistical technique for optimizing complex processes. Furthermore, this study investigated the effects of SC-CO_2_ treatment on the proximate composition and color characteristics of both the flour and LPI, as well as the nutritional quality, assessed by analyzing the fatty acid profile of the extracted oil and the amino acid content of the LPI.

## 2. Materials and Methods

### 2.1. Raw Material

For all experiments, full-fat lupin flour obtained from *Lupinus luteus* L. (Tremosilla) was used. The lupin seeds were purchased from Semillas Batlle S.A. (Barcelona, Spain), milled by an industrial vertical hammer mill (Sitem-gran Ibérica S.L., Zaragoza, Spain; 22 kW and 3 mm screen size) to finally obtain a flour with a particle size range from 200 to 1000 µm, and stored under vacuum at 20 °C until their use.

### 2.2. Experimental Design and Optimized Responses

Response surface methodology (RSM) is a powerful tool widely used for optimizing extraction processes [24,25]. RSM employs a combination of mathematical and statistical techniques to fit a polynomial equation to experimental data, effectively describing the behavior of the data set and enabling statistical predictions. Symmetrical experimental designs, such as the Box–Behnken (BB) design employed in this study, offer advantages in terms of characteristics and efficiency. Specifically, for three-variable optimization, the BB design is more economical and efficient compared to other prominent experimental designs, such as the central composite design [26]. Therefore, using a BB design in conjunction with RSM for the simultaneous optimization of the three proposed variables is an optimal approach. These designs also offer the advantage of requiring a reduced number of experiments, making them less laborious and time-consuming than other process optimization strategies [25].

**Figure 1:**
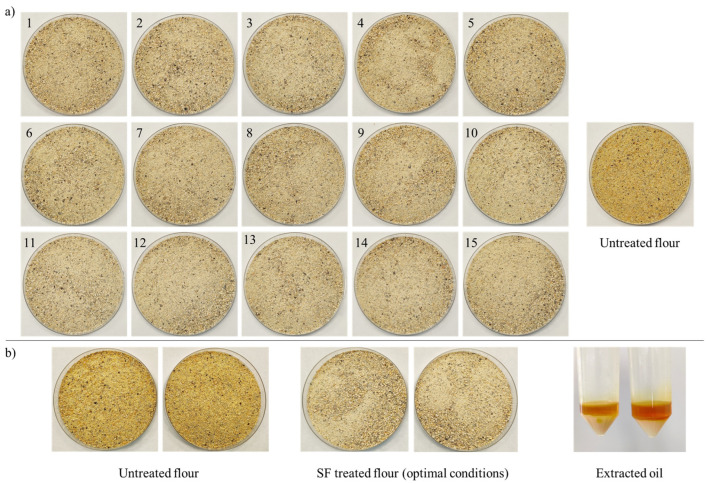
Visual aspect of the different lupin flours obtained during BBD runs (**a**) and visual aspect of lupin flour treated with supercritical CO_2_ under optimal conditions and extracted oil (**b**).

In this study, the independent variables were extraction pressure (X_1_; bar), CO_2_ flow rate (X_2_; kg/h), and extraction time (X_3_; minutes), while the dependent variables were oil reduction (y_1_) and total phenolic content (TPC) reduction (y_2_). A Box–Behnken design (3 factors at 3 levels; 3K BBD) with 15 experimental runs and three center points (3 × 1 × 15) was employed, using response surface methodology, to determine the optimal extraction conditions. Each run was performed in duplicate. Table 1 presents the Box–Behnken design (natural and coded values) of the extraction conditions and the experimental results obtained for the dependent variables. Figure 1 shows the visual appearance of the control and SC-CO_2_ treated lupin flour samples from the Box–Behnken experimental design used to optimize the extraction of anti-technological factors (ATFs).

The experimental data were fitted to a second-order polynomial equation (Equation (1)) to express the response as a function of the independent variables.

*Y* = β_0_ + β_1_*X*_1_ + β_2_*X*_2_ + β_3_*X*_3_ + β_11_*X*_1_^2^ + β_22_*X*_2_^2^ + β_33_*X*_3_^2^ + β_12_*X*_1_*X*_2_ + β_13_*X*_1_*X*_3_ + β_23_*X*_2_*X*_3_ (1)

where *Y* is the dependent variable; β_1_, β_2_, and β_3_ are the linear coefficients; β_11_, β_22_, and β_33_ the quadratic coefficients; β_12_, β_13_, and β_23_ are the interaction coefficients; while X_1_, X_2_, and X_3_ are the independent variables.

The model fitting was determined using regression coefficients and regression model, while statistical values were calculated using an ANOVA test at a 95% confidence level. The dependent variables were analyzed to obtain the optimal conditions using a multi-response surface optimization (RSM), and the optimal extraction conditions were estimated with the response desirability profiling function. The efficiency of the model was evaluated using the R^2^ value. Experimental (three extractions at the optimal conditions) and predicted values were compared based on %RSD ($\%RSD=\frac{Standard\;devidation}{Mean\;values}\times 100$) to determine the validity of the model. The model fitting, coefficient estimation, and the statistical tests of experimental design in the optimization process were performed using StatSoft STATISTICA 8.0 software (Tulsa, OK, USA).

### 2.3. Supercritical CO_2_ Treatment of Lupin Flour

A supercritical CO_2_ system (Sitec, model 101-300-AF, Zurich, Switzerland) was used for the extraction of ATFs from lupin flour. One hundred grams of lupin flour was placed in the extraction vessel, and CO_2_ (99.99%) was used as the solvent. Oil extraction was performed by pumping CO_2_ into the extraction vessel (maintained at a constant temperature of 40 °C) under varying pressure (X_1_; 300, 350, and 400 bar), CO_2_ flow rate (X_2_; 4, 6, and 8 kg/h), and extraction time (X_3_; 60, 90, and 120 min) conditions. After extraction, the vessel was gradually depressurized, and the resulting treated flour was used to determine oil and total polyphenol extraction. The extracted oil was collected in a separate vessel and recovered for fatty acid analysis, as described in Section 2.5. Both the treated flour and the extracted oil were stored at −20 °C until analysis.

### 2.4. Chemical Composition and Color Parameters

The chemical composition of both the flour and LPI was determined according to ISO methodologies: moisture content [27] (gravimetrically after oven drying at 105 °C until constant weight), protein content [28] (sample digestion followed by distillation and subsequent potentiometric titration using the Kjeldahl method, with nitrogen-to-protein conversion factors of 6.25 and 5.7), and ash content [29] (gravimetrically after calcination of organic matter in a muffle furnace at 550 °C for 8 h). Total oil content was determined using the procedure described by Domínguez et al. [30], which is based on organic solvent extraction (chloroform–methanol). Briefly, for oil extraction and quantification, 5 g of sample was mixed with 4 mL of a 1% NaCl solution, 20 mL of methanol, and 10 mL of chloroform. After homogenization for 30 s at 12,000 rpm (UltraTurrax, IKA, Barcelona, Spain), 10 mL of chloroform and 10 mL of a 1% NaCl solution were added and homogenized for 10 s. The samples were then centrifuged at 3100× *g*, and 1 mL of the lower phase (lipids and chloroform) was transferred to a pre-weighed test tube. The organic solvent was evaporated under nitrogen (1.2 bar N_2_ pressure and 50 °C; TurboVap, Biotage, Uppsala, Sweden) until dry. After cooling the tube to room temperature, it was weighed again, and the oil content was calculated by the difference in weight.

The alkaloid, saponin, and total polyphenol content (TPC) were determined using the methods described by Domínguez-Valencia et al. [24]. For alkaloid determination, 1 g of sample was initially mixed with 32 mL of 0.5 N HCl for 30 min. This extraction was repeated twice, and the combined supernatants were adjusted to pH 10 with 4 N NaOH. The alkaloids were then extracted using 50 mL of dichloromethane. After separating the organic phase by centrifugation and collection, the solvent was evaporated, and the residue was redissolved in 1 mL of methanol. Alkaloid quantification was performed by volumetric titration using 0.1% ethyl tetrabromophenolphthaleinate in ethanol as an indicator and lupanine as a standard.

For saponin extraction, 1 g of sample was mixed with 10 mL of ethanol, sonicated for 15 min, and then stirred magnetically for an additional 15 min. The solvent was evaporated, and the residue was dissolved in 10 mL of methanol. Subsequently, 0.125 mL of this methanolic solution was mixed with 1.25 mL of 72% sulfuric acid and 0.125 mL of 10% anisaldehyde in methanol. After heating at 60 °C for 1 h, the saponins were extracted with dichloromethane, and the organic phase was measured spectrophotometrically at 535 nm. Quantification was performed using an external standard method with oleanolic acid as the standard.

Total phenolic content (TPC) was determined spectrophotometrically following the procedure outlined by Singleton [31], using acidified methanol (0.2 M HCl in methanol) as the extraction solvent.

Color parameters were measured in the CIELAB color space using a portable CM-600d colorimeter (Konica Minolta Sensing Inc., Osaka, Japan). The measurements were taken with a 10° viewing angle, an 8 mm aperture size, and a pulsed xenon arc lamp filtered to illuminant D65 lighting. The colorimeter was calibrated using a white ceramic tile according to the manufacturer’s recommendations.

### 2.5. Fatty Acids

Fatty acid quantification was performed following the procedure described by Domínguez et al. [30]. Briefly, 20 mg of the extracted oil (obtained as described in the previous section) was dissolved in 1 mL of toluene and transesterified with 2 mL of 0.5 N sodium methoxide. The mixture was vortexed and allowed to stand for 15 min at room temperature. Subsequently, 4 mL of a 10% sulfuric acid solution in methanol was added and vortexed briefly. After adding 2 mL of saturated sodium bicarbonate and vortexing again, the fatty acids were extracted using 1 mL of hexane.

Methyl esters of fatty acids (FAMEs) were separated and quantified using gas chromatography with a flame ionization detector (FID) on an Agilent Technologies 7890B GC system. (Agilent Technologies, Santa Clara, CA, USA) FAME separation was achieved using a DB-23 capillary column (60 m × 0.25 mm i.d., 0.25 µm film thickness). FAME identification was based on comparisons with the retention times of standards, and quantification was performed using external standard calibration. The chromatographic conditions used were previously published [30]. Results were expressed as grams of FAME per 100 g of oil.

### 2.6. Amino Acid Content and Chemical Score

For total amino acid quantification, 100 mg of sample was hydrolyzed with 5 mL of 6 N HCl at 110 °C for 24 h. Then, 0.625 mL of the hydrolyzed sample was diluted with 25 mL of Milli-Q water (Millipore SAS, Burlington, MA, USA), and filtered. A 10 µL aliquot of the hydrolyzed amino acid extract was derivatized with 20 µL of buffer reagent and 70 µL of derivatizing reagent using the AccQ-Tag technique (Waters Corp., Milford, MA, USA), which is based on derivatization with 6-aminoquinolyl-N-hydroxysuccinimidyl carbamate. Following derivatization, the amino acids were separated and quantified using UHPLC with fluorescence detection (Acquity^®^ Arc™ UHPLC system equipped with a 2475 FLD Fluorescence Detector; Waters Corp., Milford, MA, USA). Separation was performed using an AccQ-Tag column (3 µm particle size; 3.9 × 150 mm; Waters Corp.) at 37 °C. The flow rate was 1 mL/min, and the mobile phases were as follows: (A) AccQ Tag Eluent A solution for amino acid analysis (Waters, Milford, MA, USA), (B) acetonitrile (HPLC grade), and (C) ultrapure water (Milli-Q). All liquid chromatographic conditions and the mobile phase gradient followed the procedure described by López-Fernández et al. [32]. Detection was carried out at 250 nm emission and 395 nm excitation wavelengths. Amino acid identification was based on comparisons with the retention times of standards, and quantification was performed using external standard calibration. Amino acid results were expressed as mg/g of protein.

The amino acid chemical score was calculated by comparing the essential amino acid content of the LPI to the universal reference pattern based on adult amino acid requirements [33], using Equation (2).

(2) $$Amino\;acid\;chemical\;Score\;\left(\%\right)=\frac{Essential\;amino\;acid\;insample\;\left[\frac{mg}{g\;protein}\right]}{Essential\;amino\;acid\;pattern\;concentration\;\left[\frac{mg}{g\;protein}\right]}$$

Values ≥ 100% indicated no deficiency in that amino acid.

### 2.7. Statistical Analysis

Normal distribution and homogeneity of variance were assessed using the Shapiro–Wilk test. Data for lupin flour and LPI parameters (comparing control samples with those treated under optimal SC-CO_2_ conditions) were analyzed using SPSS software (version 25.0, SPSS Inc., Chicago, IL, USA) with analysis of variance (ANOVA). The treatment effect (application of SC-CO_2_) was the fixed factor, and the studied parameters were the dependent variables. Statistical significance was determined at *p* < 0.05. Results are presented as means ± standard error.

## 3. Results and Discussion

### 3.1. Experimental Design Summary

To investigate the influence of SC-CO_2_ extraction parameters on oil and TPC reduction, response surface methodology (RSM) was employed. Optimization of the extraction process was performed by fitting second-order polynomial equations to the experimental data. The experimental results (Table 1) showed that oil reduction ranged from 38.03% to 49.85%, while TPC reduction ranged from 12.45% to 27.74%.

**Table 1:** Box–Behnken design (natural and coded values) of extraction conditions and experimental results obtained for dependent variables.

	Pressure (Bar)	Flow (kg/h)	Time (min)	Oil Reduction (%)	TPC Reduction (%)
	Factors			Response Variables	
Run	x_1_	x_2_	x_3_	y_1_	y_2_
1	300 (−1)	4 (−1)	90 (0)	45.89 ± 2.40	16.64 ± 8.87
2	400 (1)	4 (−1)	90 (0)	46.70 ± 5.33	27.74 ± 5.30
3	300 (−1)	8 (1)	90 (0)	42.59 ± 5.91	23.51 ± 3.54
4	400 (1)	8 (1)	90 (0)	44.74 ± 3.64	21.93 ± 16.74
5	300 (−1)	6 (0)	60 (−1)	39.26 ± 5.34	18.29 ± 6.56
6	400 (1)	6 (0)	60 (−1)	42.28 ± 6.50	15.98 ± 1.73
7	300 (−1)	6 (0)	120 (1)	45.89 ± 4.03	17.42 ± 11.98
8	400 (1)	6 (0)	120 (1)	45.11 ± 5.20	21.00 ± 9.00
9	350 (0)	4 (−1)	60 (−1)	38.03 ± 6.36	20.04 ± 2.74
10	350 (0)	8 (1)	60 (−1)	40.99 ± 9.62	12.45 ± 0.29
11	350 (0)	4 (−1)	120 (1)	40.83 ± 8.06	13.13 ± 6.09
12	350 (0)	8 (1)	120 (1)	43.53 ± 4.42	18.77 ± 0.83
13	350 (0)	6 (0)	90 (0)	40.58 ± 5.65	19.04 ± 0.95
14	350 (0)	6 (0)	90 (0)	39.79 ± 4.90	27.18 ± 2.75
15	350 (0)	6 (0)	90 (0)	49.85 ± 3.48	16.55 ± 6.04

TPC: total polyphenol content.

Using these values, regression analysis and ANOVA were performed to establish the functional relationships for approximating and predicting the responses (Table 2). The coefficients indicated reasonable model accuracy (R^2^ = 0.5751 for oil reduction and R^2^ = 0.7318 for TPC reduction), suggesting a correlation between the model and the experimental data.

The ANOVA results indicated that none of the individual parameters (pressure, CO_2_ flow rate, and time) had a statistically significant effect on either oil or TPC reduction. The linear, quadratic, and interaction coefficients of the independent variables also showed no statistically significant relationship (*p* > 0.05) with the experimental data. However, the lack-of-fit test (*p*-value 0.88853 for oil reduction and *p*-value 0.93153 for TPC reduction) was non-significant, which supports the model’s adequacy in representing the experimental data for all variables.

The lack of significance observed for the optimized parameters could be attributed to the variability observed during the extraction of oil and polyphenols. In the case of TPC specifically, the high variability may be related to the low specificity of extraction with pure CO_2_, which has low polarity and thus a limited capacity to extract these compounds. This could increase the variability between different extractions within the Box–Behnken design, consequently masking any significant influence of the individual parameters.

**Figure 2:**
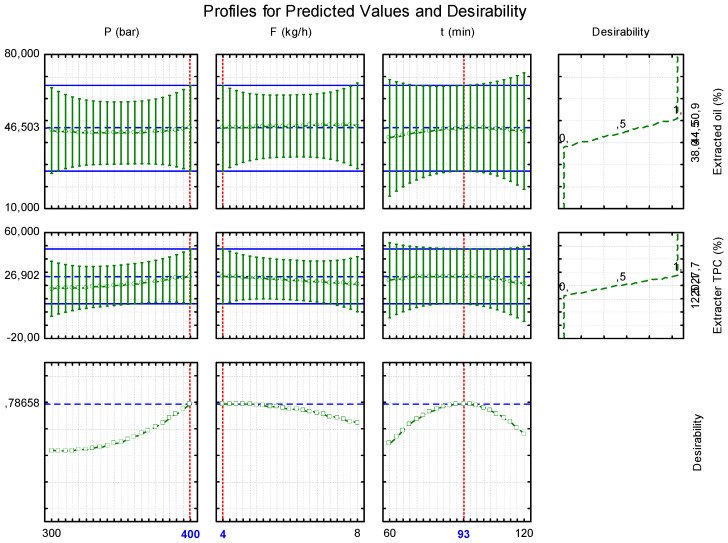
Profiles for predicted values and desirability.

It is well established that several factors, including extraction pressure and CO_2_ flow rate, influence the efficiency of oil extraction during SC-CO_2_ treatment [23]. In this study, although no statistically significant influence of the studied variables was observed, the predicted value profiles (Figure 2) suggest that desirability increased with increasing pressure, reaching a maximum at 400 bar. Conversely, CO_2_ flow rate had the opposite effect, with the lowest flow rate (4 kg/h) exhibiting the highest desirability. Finally, extraction time showed a progressive increase in desirability between 60 and 93 min, followed by a decrease after 93 min.

Similar trends have been reported by other researchers, who observed increased soybean oil yield with increasing SC-CO_2_ extraction pressure (from 300 to 400 bar) [23]. Under isothermal conditions, increasing pressure leads to an increase in CO_2_ density, thus enhancing its solvent power [34], which explains the effect of pressure on the extractability of oil and TPC. In this study, all extractions were performed at 40 °C. The CO_2_ density at 300 bar was 964.33 ± 2.08 kg/m^3^, which significantly increased to 975.33 ± 3.78 kg/m^3^ at 350 bar and further to 987.67 ± 6.11 kg/m^3^ at 400 bar. This increase in density contributes to the enhanced solvation power for apolar and medium-polar molecules [34]. Consequently, the higher CO_2_ density at 400 bar extraction pressure promotes mass transfer between the lupin flour and the CO_2_, improving the extraction of soluble compounds. This effect is attributed to the increased solvent power of the CO_2_, which facilitates matrix swelling, solute solvation, and greater solubility in the fluid phase [35,36].

Reducing the CO_2_ flow rate increased ATF extraction, likely because solvation at high CO_2_ flow rates is lower than at low flow rates, suggesting that the highest extraction of anti-technological compounds occurred at 4 kg/h. This highlights the importance of balancing ATF solubility in CO_2_ and its diffusion during extraction. The relatively polar nature of the highly unsaturated oil in lupin seeds likely contributed to its greater extraction at lower flow rates, as this provided sufficient contact time between the flour and the CO_2_ for effective mass transfer [36]. Therefore, the optimal flow rate likely lies in the region where both solubility and diffusion play significant roles [37].

**Figure 3:**
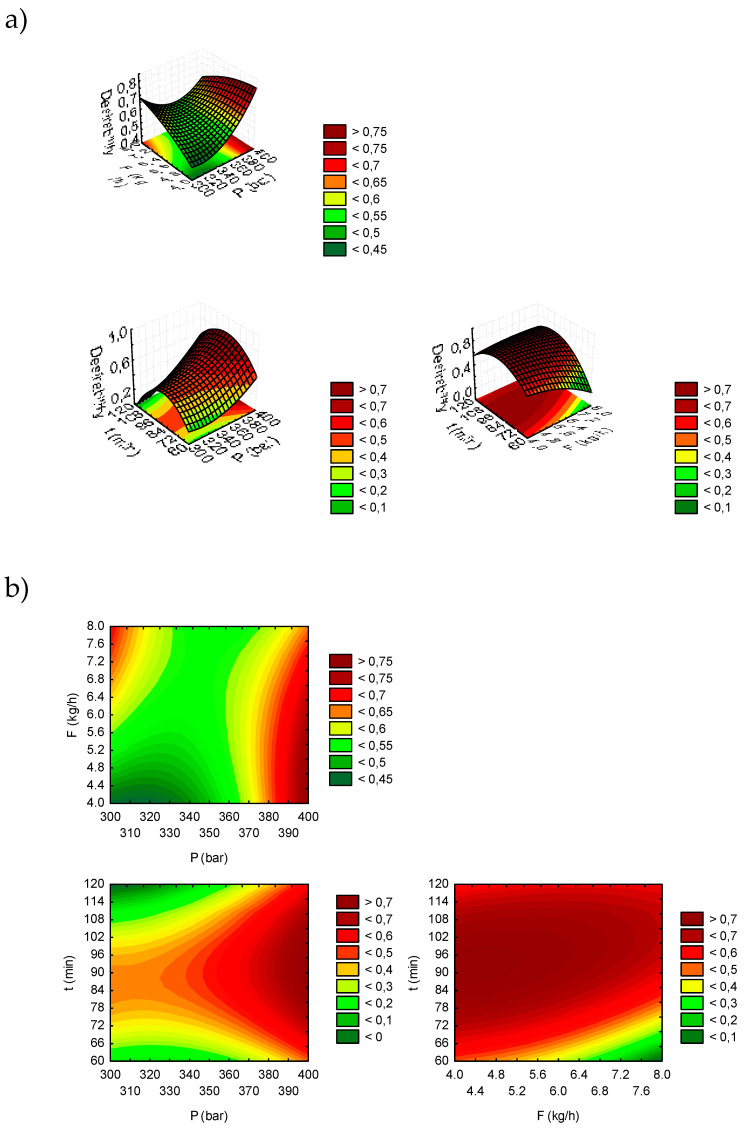
Response surface plots (**a**) and contour plots (**b**) (desirability) as function of pressure, flow, and extraction time.

Regarding polyphenols, the use of pure CO_2_ had a limited impact on their extraction. The low dielectric constant of CO_2_ explains its poor ability to extract polar molecules [34]. Indeed, several studies have suggested using co-solvents to increase polarity and improve the extraction of phenolic compounds [38]. For example, one study examining germinated and ungerminated *L. luteus* seeds treated with SC-CO_2_ for polyphenol extraction [7] concluded that modifying the SC-CO_2_ with 16% ethanol as a co-solvent positively influenced the solvating properties of the CO_2_ and increased phenolic extraction. In contrast, the present study used pure CO_2_ (without a co-solvent), which resulted in low extractability of these polar phenolic compounds. The optimal SC-CO_2_ conditions, determined using response desirability and visualized in surface (Figure 3a) and contour (Figure 3b) plots, indicated that maximizing ATF reduction was achieved at 400 bar pressure, a 4 kg/h CO_2_ flow rate, and a 93 min extraction time. The desirability obtained under these conditions was 0.7866. The predicted values at these optimal conditions were a 46.50% reduction in oil and a 26.90% reduction in TPC.

The RSM model was validated by comparing the predicted and experimental values (Table 3). For oil reduction, the predicted (46.5%) and experimental (42.39%) values were in close agreement. However, the experimental value for TPC reduction (40.67%) was notably higher than the predicted value (26.9%). The relative standard deviation (%RSD) was 6.54% for oil reduction and 28.82% for TPC reduction. These results suggest that the model is a reliable tool for predicting oil reduction in SC-CO_2_ extractions, exhibiting a coefficient of variation below 10%. However, the model is less reliable for predicting TPC reduction, and it cannot be used for the TPC reduction.

### 3.2. Effect of Supercritical CO_2_ Treatment on Lupin Flour Characteristics

#### 3.2.1. Chemical Composition, Total Polyphenols Content, and Color Parameters

The chemical composition and color parameters of treated and untreated lupin flour are presented in Table 4. Among all nutrients, protein and fat content are the most critical parameters in flour, as they play a vital role in LPI quality and functionality [20]. The flour yield after SC-CO_2_ treatment was 92.76%, which can be attributed to the removal of 46.5% of the initial oil and the partial removal of moisture, as discussed below.

In this study, SC-CO_2_ treatment did not significantly affect protein content, which ranged from 42.82 to 43.57 g/100 g (N × 5.7; 39.06 and 39.74 g/100 g) in the control and treated flour, respectively. While a slight increase was observed, it was not statistically significant. Conversely, other studies have reported that oil removal leads to a significant increase in protein content in both soy flour [11,21,22,39] and lupin flour [18,35]. The fact that some of these studies reported composition on a fresh matter basis, while the present study uses dry matter, could partially explain these differences. The ash content significantly increased after SC-CO_2_ extraction, from 3.96 to 4.24 g/100 g. This is likely due to the removal of oil, which results in a higher proportion of ash in the flour dry matter. This observation is consistent with the findings of Kang et al. [11,21] and Shin et al. [39], who reported that SC-CO_2_ treatment increased the proportion of ash in soy flour. An increase in ash content was also observed with SC-CO_2_ treatment of *L. mutabilis* flour [35].

The chemical composition of the control flour was similar to that reported for *L. mutabilis* by other researchers (approximately 11% moisture, 15% fat, and 44% protein) [8]. Other studies on *L. mutabilis* flour reported 6.33% moisture, 15% fat, and 42.23% protein. In full-fat *L. luteus* flour, protein content has been reported to be around 42% [5,17], oil content ranged from 3.79% to 6.06% [5,17,40], and ash content from 4.05% to 4.90% [17,40]. Another study found that full-fat flour from *L. albus* and *L. angustifolius* contained 41–43% protein (37–39% N × 5.7), while fat content varied between 5.8% in *L. angustifolius* and 11.9% in *L. albus* [12]. This highlights the strong influence of lupin species on flour composition, with oil being one of the most variable components, consequently affecting the levels of other nutrients. Indeed, oil content in *L. albus*, *L. angustifolius*, and *L. luteus* can vary between 4% and 12% [41]. However, the values obtained in this study for *L. luteus* are consistent with previously reported data for the same species.

As expected, the apolar nature of CO_2_ facilitated the extraction of lipophilic compounds, including oil and lipid-soluble pigments (primarily carotenoids). In this study, the oil content of the control flour (6.54 g/100 g) was reduced to 3.83 g/100 g after SC-CO_2_ extraction. The resulting extract showed a clear separation into a lipophilic phase (with an intense orange color) and a hydrophilic phase (Figure 1). The CO_2_ extracted both oil and lipophilic compounds, and also a portion of the moisture content. This explains not only the oil reduction but also the significant decrease in moisture. Water has a small finite solubility in SC-CO_2_ [37], which explains the co-extraction of oil and water during SC-CO_2_ treatment. Similar results were observed in soy flour, where defatting with SC-CO_2_ significantly decreased both fat (18.78% vs. 1.64% in control and treated flour, respectively) and moisture (7.23% vs. 5.69% in control and treated flour, respectively) [11,22]. Another study reported that high-pressure (300 bar) SC-CO_2_ extraction promoted the removal of both oil and moisture in soy flour compared to both untreated flour and low-pressure (100 bar) SC-CO_2_ extraction [21]. In *L. mutabilis* flour, SC-CO_2_ treatment also resulted in significantly lower values for both fat (14.81% vs. 15.71%) and moisture (4.29% vs. 6.33%) compared to untreated flour [35].

The use of SC-CO_2_ extraction has proven effective in removing oil from various matrices, including soybean okara [23], soy flour [11], and lupin flour [3,20,35]. The appearance of the extracted oil (Figure 1), similar to that reported in other studies [23], suggests the co-extraction of carotenoids and other lipophilic coloring agents along with the oil. Therefore, in summary, the reduction of both moisture and oil during SC-CO_2_ treatment resulted in significant changes in lupin flour chemical composition, with the exception of protein content.

The difference in oil reduction between this study (42%) and our previous work (66.2%) where SC-CO_2_ was applied directly to LPI [24] can be attributed to several factors. First, anti-technological factors are located within the cells [9]. In lupin flour, where cells are partially intact, CO_2_ must cross the cell membrane to solubilize and extract the oil. However, when SC-CO_2_ is applied to LPI, where cells have been disrupted and the proteins extracted and “purified” (with the carbohydrate fraction removed), the oil and other ATFs are released and much more accessible. This facilitates their extraction and diffusion into the CO_2_, explaining the greater removal of polar compounds in the latter case. Second, the particle size of lupin flour (207 µm) is considerably larger than that of LPI (41.7 µm), resulting in a smaller specific surface area for the flour (177.5 m^2^/kg) compared to the LPI (421.3 m^2^/kg). This difference enhances CO_2_ diffusion through the LPI, significantly improving ATF extraction. It is well established that oil extraction is inversely related to particle size, as larger particles hinder oil mass transfer [36]. Finally, the higher moisture content of the flour (6.89 g/100 g) compared to the lyophilized LPI (<0.1 g/100 g) likely hinders SC-CO_2_ penetration, limiting the extraction of lipophilic compounds. Water affects surface tension and contact angle, reducing the access of SC-CO_2_ to internal pores and increasing the mass transport path [37]. Therefore, the observed difference in oil reduction is expected, as SC-CO_2_ application directly to the LPI is inherently more effective than its application to the flour.

The total polyphenol content (TPC) in the control flour was 225 mg GAE/100 g, decreasing to 135.9 mg GAE/100 g in the SC-CO_2_-treated flour. The TPC value of the control flour is consistent with values reported for *L. luteus* flour by other researchers (272 mg GAE/100 g) [5]. The TPC reduction (39.5%) observed with the SC-CO_2_ procedure aligns with the value obtained during the optimization process (40.67%). As previously mentioned and discussed (Section 3.1), the use of pure CO_2_ without co-solvents limits the extraction of polyphenols [38], explaining these results.

The color parameters reflected the typical yellow color of lupin flour, characterized by high luminosity (L*) and yellowness (b*) values, and low redness (a*) values [17,42]. In this study, the L* (77–79), b* (23–31), and a* (1.72–3.08) values for both treated and untreated samples were close to those reported for full-fat *L. luteus* flour (L* 77.59; a* 2.76; b* 31.92) by other authors [17]. SC-CO_2_ treatment did not affect luminosity but significantly decreased redness (3.08 vs. 1.72 in control and treated flour, respectively) and yellowness (31.88 vs. 23.09 in control and treated flour, respectively). This agrees with studies on soy flour, where the defatting step, which removes carotenoids, resulted in a significant reduction in both yellowness and redness [39]. These color changes can be attributed to variations in lipophilic pigments, mainly carotenoids [17,20,24]. Significant levels of lutein, zeaxanthin, α-carotene, and β-carotene have been reported in lupin seeds and their oils [8,43,44]. During SC-CO_2_ extraction, these pigments are co-extracted with the lupin oil, leading to a significant decrease in a* and b* values. This co-extraction also explains the intense yellow color of the extracted oil (Figure 1).

#### 3.2.2. Fatty Acids Profile

Lupin oils offer several health benefits for humans [43]. Therefore, the oil fraction recovered after SC-CO_2_ extraction, rich in active and nutritious compounds such as unsaturated fatty acids, can be utilized as an alternative and non-traditional source of healthy and bioactive compounds. Utilizing this fraction also contributes to a circular economy. Furthermore, the SC-CO_2_ extraction method helps preserve the oil’s high quality and nutrient content [23].

In this study, the predominant fatty acids were oleic and linoleic acid, present in similar amounts (26–27 g/100 g oil), followed by behenic acid (5.9 g/100 g oil), palmitic acid (5.33 g/100 g oil), and α-linolenic acid (5.32 g/100 g oil) (Table 5). These fatty acids constituted over 75% of the lupin oil and more than 85% of the total fatty acids, consistent with previous findings [17]. This profile indicates that lupin oil is a valuable source of essential fatty acids (linoleic and α-linolenic acids) and has a balanced n-6/n-3 PUFA ratio (4.89), aligning with recommendations for a healthy diet [45]. Similar ratios have been observed in *L. albus* [46] and *L. luteus* [17,44]. The total unsaturated fatty acid content was approximately 63 g/100 g oil, with roughly equal proportions of MUFAs and PUFAs (approximately 31 g/100 g oil each). This lipid profile, characterized by high levels of balanced unsaturated fatty acids, is associated with preventing chronic and coronary heart disease risk and reducing serum cholesterol, LDL cholesterol, and triglycerides [47]. Total fatty acids accounted for 81.25% of the oil fraction.

Other researchers have reported similar fatty acid profiles in lupin flour and/or oils, where oleic and linoleic acids are the predominant fatty acids, and significant amounts of α-linolenic acid are also present [7,8,17,43,46,48]. Siger et al. [44] and Spina et al. [48] also reported high levels of behenic and palmitic acids in *L. luteus* seeds, with values comparable to those found in this study.

Furthermore, the fatty acid profile observed in this study is consistent with previous findings for the same species (*L. luteus*) [17], suggesting that the SC-CO_2_ technique (at the applied temperature and pressure) did not alter the fatty acid profile or the content of essential fatty acids. Based on its fatty acid composition, the lupin oil obtained in this study can be considered to have a healthy profile, characterized by high levels of oleic acid and essential fatty acids (linoleic and α-linolenic acids), as well as a balanced proportion of PUFAs.

### 3.3. Effect of Supercritical CO_2_ Treatment on Lupin Protein Isolate Characteristics

#### 3.3.1. Extraction Yield, Chemical Composition, Alkaloids, Saponins, and Color Parameters

The total yield was not affected by the treatment (approximately 24% in both cases), but the protein yield significantly increased (*p* < 0.05) in the LPI-SF (56.95%) compared to the LPI-control (53.29%). The total yield values are consistent with those reported in previous studies on *L. luteus* (22.37–23.19 g LPI/100 g flour) [5,17]. Furthermore, as observed in this research, Nahimana et al. [14] reported that the defatting process increased protein yield during LPI extraction. This increase could be attributed to the lower residual oil content in the defatted flour, which facilitates protein extraction during alkaline solubilization.

The protein purity of the LPI-control (87.42%) is consistent with values reported in previous studies, which found protein content between 86.3% and 88.58% in LPI obtained from *L. luteus* [5,17,24]. The oil (8.31–14.34%) and ash (4.77–6.69%) contents also align with values observed in other LPIs (5.27–15.6% fat and 3.18–6.41% ash) obtained from *L. luteus* flour using alkaline solubilization–isoelectric precipitation [5,17,24].

The higher protein content (purity) of the LPI-SF compared to the LPI-control highlights the importance of the preceding SC-CO_2_ treatment step, which effectively removed potentially co-extracted compounds. The increased protein purity in the LPI-SF can likely be attributed to both the lower residual oil content and the removal of other lipophilic compounds from the flour by SC-CO_2_. The ash content also increased in the LPI-SF, likely because ash contributes proportionally more to the dry matter after oil removal. These results are consistent with those observed in a previous study, where flour defatting significantly increased the purity of the lupin protein isolate [14].

Consistent with our findings, LPI obtained from defatted *L. albus* and *L. angustifolius* flour also exhibited higher protein content (92–94%) compared to LPI from full-fat flour (87–91%) [12]. Direct SC-CO_2_ defatting of LPI also resulted in a significant increase in protein purity (96% vs. 86.3%) [24]. Vogelsang-O’Dwyer et al. [3] found that LPI obtained from SC-CO_2_-defatted *L. albus* and *L. angustifolius* flours had protein contents of 92.6–94.4%, respectively. Another study reported that defatted *L. campestris* flour yielded an LPI (using alkaline extraction–isoelectric precipitation) with 93.2% protein purity [49], which aligns with the data obtained in this study. Furthermore, the residual oil content in the LPI also significantly decreased, from 11–12% to approximately 3%, in LPI obtained from defatted flours [12]. Due to oil removal from the lupin flour after SC-CO_2_ treatment, the corresponding LPI is expected to have higher protein and ash content and lower oil content.

On the other hand, alkaline solubilization can promote oil saponification, increasing the oil’s solubility in the aqueous phase [10]. This oil is then co-precipitated with the proteins and concentrated during dehydration [10,17]. This is the primary reason for the higher oil content (approximately a 117% increase) observed in LPI compared to lupin flour. This same trend has been reported by several authors, who observed a 46% [17], or 172% [5] increase in lipids in isolates compared to the corresponding flour.

The bitter taste of lupin is associated with its high alkaloid content [49]. In this study, SC-CO_2_ treatment significantly (*p* < 0.05) reduced alkaloid content in the LPI (from 607 to 388 mg/kg). However, the use of an apolar solvent like CO_2_ was not highly effective in removing alkaloids, as they are water-soluble compounds [6,49]. This result aligns with findings by Rosas-Quina et al. [35], who noted that the apolar nature of SC-CO_2_ limited the extraction of highly polar lupin alkaloids. Similarly, using SC-CO_2_ for LPI functionalization from *L. luteus* did not significantly alter alkaloid content (approximately 660 mg/kg) [24]. The greatest reduction in alkaloids occurred during LPI extraction, where alkaline extraction, acid precipitation, and washes before lyophilization reduced their levels from 3864 ± 735 mg/kg in lupin flour to approximately 500 mg/kg in the LPI. Lupanine is the major alkaloid in lupin, although other minor quinolizidine alkaloids are also present [6]. The recommended maximum limit for alkaloids is 200 mg/kg, as higher levels can cause intoxication [5,35]. In this study, while alkaloids were reduced during LPI extraction and SC-CO_2_ treatment, their content in the final LPI still exceeded recommended limits, necessitating additional processing to ensure they are not toxic to humans. Saponin content also slightly decreased during SC-CO_2_ treatment (from 1.03 to 0.86 g oleanolic acid/100 g). The saponin values in the LPI-control were similar to those reported for *L. luteus* flour (1.99 g oleanolic acid/100 g) by other authors [5]. However, the effect of SC-CO_2_ on saponin extraction was not statistically significant (*p* > 0.05). A previous study [24] observed a similar (but significant) saponin reduction (from 0.99 to 0.86 mg oleanolic acid/100 g) in LPI treated with SC-CO_2_. Like alkaloids, saponins are highly soluble in polar solvents, such as water or alcoholic solutions, but insoluble in apolar solvents [50], which explains the limited effect of SC-CO_2_ extraction on their levels. Saponins can form complexes with minerals and vitamins, reducing their bioavailability and the nutritional quality of LPI [5]. Additionally, saponins can reduce protein solubility and increase surface hydrophobicity and foaming capacity of protein isolates, thus affecting their functional properties [51]. Therefore, a debittering process to remove alkaloids from LPI is necessary and can also remove other ATFs such as saponins [46].

Considering the results presented, it is evident that applying SC-CO_2_ to lupin flour shows promise as a strategy for purifying both the flour and derived protein isolates. It also offers a method for obtaining an oil rich in bioactive substances with potential applications in the food industry. However, this study also highlights limitations of SC-CO_2_ technology, notably its limited capacity to extract other ATFs, such as polyphenols, saponins, and alkaloids. Therefore, for lupin specifically, the use of co-solvents to increase polarity could improve the extraction of these ATFs, making the process more suitable for industrial scale-up.

**Figure 4:**
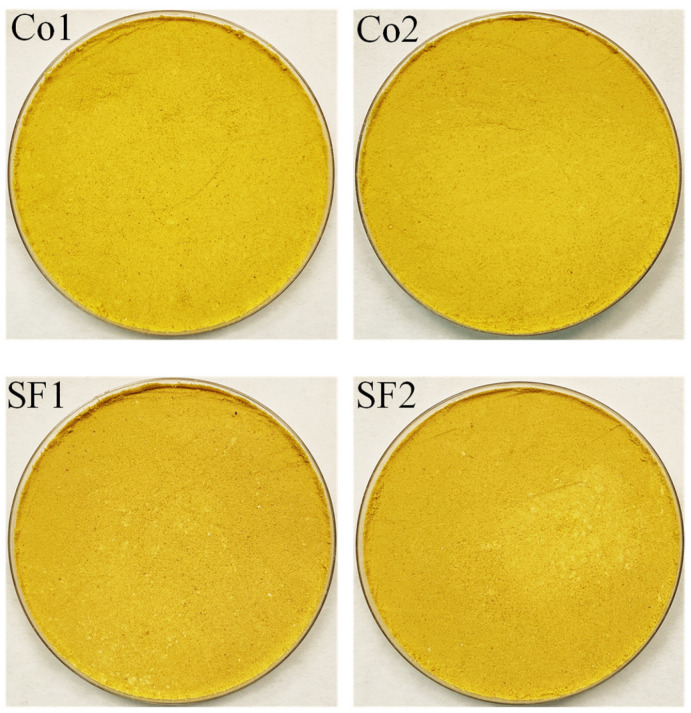
Visual aspect of lupin protein isolate obtained from untreated flour (Co1 and Co2) and from supercritical CO_2_-treated flour under optimal conditions (SF1 and SF2).

Regarding color parameters (Table 6), LPI exhibited a more intense yellow-orange hue than the flour. This was expected due to the higher lipid content in LPI, which leads to a greater concentration of lipophilic pigments. The LPI color (Figure 4; Table 6) showed similar values to those reported for other lupin protein isolates [17,24]. Other researchers reported significantly higher redness (8.59) and yellowness (64.16), and lower luminosity (66.64) values than those obtained in this study [5].

Variations in pigment levels, lupin species, extraction conditions, and drying procedures can explain these differences between studies. The LPI-SF had significantly lower redness (4.45 vs. 5.10) and yellowness (42.81 vs. 47.66) than the LPI-control, while luminosity remained relatively unchanged. This is expected, as the removal of both oil and lipophilic pigments during SC-CO_2_ extraction leads to a decrease in the a* and b* color parameters. The lower oil content of the LPI-SF also implies a lower fat-soluble pigment content compared to the LPI from untreated flour, explaining these color variations. The extracted oil (Figure 1) clearly exhibited an intense yellow color, consistent with lupin oil obtained in a previous study [24]. This is due to the high concentration of pigments extracted from the flour during SC-CO_2_ treatment, which are consequently absent in both the treated flour and the resulting LPI. Similar observations have been previously reported, where SC-CO_2_ treatment of lupin protein isolate resulted in an increase in L* and a decrease in a* and b* values [24]. Similarly, tofu made from SC-CO_2_-treated soy flour also showed lower a* and b* values, which was attributed to the defatting process and the removal of carotenoids and other color compounds from the soy flour [21,22]. Therefore, it is clear that SC-CO_2_ treatment not only improves the flour quality but that these improvements are carried over to the products subsequently derived from the treated flour.

#### 3.3.2. Amino Acid Composition and Amino Acid Chemical Scores

Given the global shortage of high-quality protein [8], utilizing lupin protein isolates presents a promising opportunity to address this issue. Lupin species are known for their characteristic amino acid profile, which is well balanced and includes significant amounts of essential amino acids [6,8]. Both the quantity and quality of protein are crucial for food applications of protein isolates, particularly as ingredients in both traditional and novel foods [52]. Therefore, analyzing the amino acid composition (Table 7) and calculating the chemical score (Table 8) are essential steps in demonstrating the value of lupin protein isolate for food fortification and its potential health benefits for humans.

In this study, the most abundant amino acid was glutamic acid (approximately 235 mg/g protein), followed by arginine and aspartic acid, each present at approximately 110 mg/g protein. Significant levels of essential amino acids were also observed, including leucine (82 mg/g protein), lysine (57 mg/g protein), isoleucine (45 mg/g protein), phenylalanine (41 mg/g protein), valine (39 mg/g protein), and threonine (35 mg/g protein). These results align with amino acid profiles reported for various lupin species, including *L. albus* [3,6,12], *L. angustifolius* [3,6,12,15,18], *L. luteus* [6,17], *L. campestris* [49], and *L. mutabilis* [6]. Consistent with previous reports [49], the LPI exhibited low methionine content. Similar to other LPIs [6,17,24], the lowest amino acid levels were observed for methionine (approximately 4 mg/g protein) and cysteine (approximately 18 mg/g protein). The essential amino acid content reached 32–33%, which is typical for *L. luteus* [17,24], a species known to have a higher proportion of essential amino acids than other lupin species [6].

In this study, the amino acid profiles of the control and SC-CO_2_-treated LPI were very similar, with significant differences observed in the levels of only a few amino acids, though the overall values remained comparable. This similarity could be explained by the fact that proteins are not extracted during SC-CO_2_ treatment and therefore remain largely unaltered. Similar results were reported when SC-CO_2_ extraction was applied to *L. luteus* LPI, where the treatment had minimal impact [24]. Since the rest of the LPI production process was identical for both samples, it would not be expected to influence amino acid content. This has been confirmed in previous studies, where researchers observed no or only minor changes in amino acid levels after various processing conditions [12], demonstrating the stability of amino acid profiles and content in protein isolates. 

The primary limiting amino acids in legumes are sulfur-containing amino acids. Lupin is typically deficient in methionine and cysteine [3,10,12]. In this study, the LPI was only deficient in methionine (approximately 23%), while the cysteine chemical score was about 300%. Similar scores were reported in previous studies on *L. luteus* LPI [17,24]. Other researchers reported Met + Cys scores of 62% and 66% in *L. albus* and *L. angustifolius*, respectively [3], which are lower than the values obtained in this *L. luteus* study. This is expected, as legume proteins generally have low levels of sulfur-containing amino acids [3]. Some studies have also indicated deficiencies in threonine, lysine, and valine in lupin species [3,6,15]. However, this research demonstrates that the LPI content of these amino acids meets FAO/WHO/UNU [33] requirements (100%, 124%, and 152% for valine, lysine, and threonine, respectively). This difference may be attributed to *L. luteus* having higher levels of these essential amino acids than other lupin species [6].

Another important protein quality index is the total indispensable amino acid content. A high-quality protein for human consumption should have an essential amino acid index greater than 277, according to recommended guidelines. In this study, both LPI samples significantly exceeded this recommendation, with values around 375. This demonstrates that, regardless of processing (with or without SC-CO_2_ treatment), the protein isolate obtained from *L. luteus* possesses high nutritional value and provides a substantial supply of essential amino acids. Similar results, with total indispensable amino acid values reaching approximately 400, have been reported in other LPI studies [24].

While SC-CO_2_ treatment had a limited influence on the amino acid chemical score, with only lysine and methionine scores showing slight increases, the total indispensable amino acid content was marginally higher in the LPI-control than in the LPI-SF. However, these values were very similar between the two samples (373–381). Although statistically significant (*p* < 0.05) differences were observed, the magnitude of these differences was minimal and primarily due to the small variations in amino acid content between the control and SF LPI. A previous study also reported that supercritical CO_2_ treatment did not cause significant changes in either the amino acid profile or the chemical scores [24], which is consistent with the findings of this study. Therefore, it can be concluded that SC-CO_2_ treatment had limited to no impact on the protein quality (amino acid levels and scores) of lupin protein isolates derived from *L. luteus*.

## 4. Conclusions

The results indicate that supercritical carbon dioxide (SC-CO_2_) extraction is a valuable technique for improving lupin protein isolate (LPI) purity. This method effectively reduces the levels of anti-technological factors, mainly oil content. This process also facilitates the co-extraction of lipophilic pigments, enhancing the color of both lupin flour and the resulting LPI. The LPI derived from SC-CO_2_-treated flour exhibited superior nutritional properties, characterized by higher protein purity compared to LPI from untreated flour. However, a crucial next step involves implementing a debittering process to effectively remove alkaloids and other ATF from the final LPI product.

Beyond the protein fraction, the extracted oil also holds valuable potential for incorporation into the food industry, promoting a circular economy approach. This oil is rich in phytonutrients and its fatty acid profile suggests its suitability as a novel source of healthy and balanced dietary fat.

The findings and conclusions of this study offer valuable insights for the food industry in designing optimized lupin-based food processing systems. Practically, this SC-CO_2_ procedure enables solvent-free flour treatment, leading to reductions in processing time, waste generation, and energy consumption. While SC-CO_2_ proves to be a selective and efficient method for removing non-polar molecules and improving LPI quality, further research and optimization are needed to enhance the extraction of specific ATF, particularly alkaloids. Finally, the composition of the SC-CO_2_-treated LPI suggests its potential for use in the formulation and fortification of novel food products, presenting significant opportunities within the food industry.

## Figures and Tables

**Table 2:** Regression coefficients of the second-order polynomial equation and statistical parameters (ANOVA) of the model.

	Oil Reduction		TPC Reduction	
	Regression Coefficient	*p*-Value	Regression Coefficient	*p*-Value
Mean/Interc. (β_0_)	44.96923	0.0044 ***	20.92404	0.0002 ***
Linear				
Pressure (β_1_)	0.81064	0.7027	1.34624	0.3682
Flow (β_2_)	0.37216	0.8585	−0.11310	0.9370
Time (β_3_)	2.37094	0.3268	0.44643	0.7563
Crossed				
(β_12_)	0.20355	0.9448	−3.17221	0.1604
(β_13_)	−0.96205	0.7473	1.47253	0.4789
(β_23_)	−0.13555	0.9632	3.30293	0.1469
Quadratic				
Pressure (β_11_)	1.63761	0.6071	1.80473	0.4092
Flow (β_22_)	−0.46632	0.8792	−0.27411	0.8966
Time (β_33_)	−2.70704	0.4231	−4.54785	0.0725
Model Statistics				
Lack of Fit (*p*-value)	0.88853 (^ns^)		0.93153 (^ns^)	
R^2^	0.5751		0.7318	
Pure error	27.12484		30.87594	

TPC: total polyphenol content; ***: significant parameter (*p* < 0.001); (^ns^): not significant.

**Table 3:** The response of predicted and experimental values of the optimized conditions.

Response	Predicted Values	Experimental Values	%RSD
Oil reduction (%)	46.50	42.39 ± 1.45	6.54
TPC reduction (%)	26.90	40.67 ± 2.39	28.82

TPC: total polyphenol content.

**Table 4:** Effect of supercritical CO_2_ treatment (under optimal conditions) on chemical composition, total polyphenols content, and color parameters lupin protein flour.

	Flour Type		Sig.
	Control	SF	
Flour yield (%)	-	92.76 ± 2.08	-
**Chemical composition (g/100 g flour)**			
Moisture	6.89 ± 0.01	5.24 ± 0.08	***
Oil ^†^	6.54 ± 0.11	3.83 ± 0.09	***
Protein (N × 6.25) ^†^	42.82 ± 0.33	43.57 ± 0.98	ns
Protein (N × 5.7) ^†^	39.06 ± 0.31	39.74 ± 1.05	ns
Ash	3.96 ± 0.03	4.24 ± 0.02	***
Total polyphenols content (mg GAE/100 g)	225.04 ± 11.24	135.88 ± 5.42	***
**Color parameters**			
L*	77.64 ± 0.13	79.60 ± 2.42	ns
a*	3.08 ± 0.33	1.72 ± 0.53	*
b*	31.88 ± 0.50	23.09 ± 0.50	***

Sig: significance; ns: not significant; *: *p* < 0.05; ***: *p* < 0.001; ^†^: results expressed as g/100 g of dry matter.

**Table 5:** Fatty acids profile (g/100 g oil) of lupin oil extracted with supercritical CO_2_ treatment.

	Extracted Oil		
Fatty Acids	Mean ± SD	Min	Max
C14:0	0.21 ± 0.01	0.20	0.22
C16:0	5.33 ± 0.17	5.24	5.59
C18:0	2.67 ± 0.07	2.63	2.78
C18:1n-9	27.76 ± 0.77	27.28	28.90
C18:1n-7	0.54 ± 0.02	0.51	0.56
C18:2n-6	25.98 ± 0.73	25.55	27.07
C18:3n-3	5.32 ± 0.16	5.24	5.56
C20:0	2.86 ± 0.06	2.81	2.94
C20:1n-9	1.84 ± 0.04	1.81	1.89
C20:2n-6	0.18 ± 0.01	0.17	0.19
C21:0	0.19 ± 0.00	0.19	0.20
C22:0	5.90 ± 0.11	5.80	6.03
C22:1n-9	0.84 ± 0.01	0.83	0.86
C22:2n-6	0.18 ± 0.04	0.15	0.24
C23:0	0.26 ± 0.01	0.25	0.27
C24:0	0.82 ± 0.01	0.80	0.84
SFA	18.43 ± 0.42	18.11	19.04
MUFA	31.10 ± 0.85	30.53	32.35
PUFA	31.73 ± 0.94	31.18	33.13
n-3	5.39 ± 0.16	5.30	5.63
n-6	26.34 ± 0.77	25.88	27.49
Total	81.25 ± 2.20	79.81	84.51

SFA: saturated fatty acids; MUFA: monounsaturated fatty acids; PUFA: polyunsaturated fatty acids. In the table only the fatty acids that represented more than 0.1% of the total fatty acids are presented, although all the identified fatty acids have been used for the calculation of SFA, MUFA, and PUFA.

**Table 6:** Effect of supercritical CO_2_ treatment on lupin protein isolate yields, chemical composition, alkaloids, saponins, and color parameters.

	LPI Type		Sig.
	LPI-Control	LPI-SF	
Total yield (g LPI/100 g of flour)	24.30 ± 0.97	24.21 ± 0.26	ns
Protein yield (%)	53.29 ± 2.37	56.95 ± 0.45	*
**Chemical composition (g/100 g)**			
Moisture	0.00 ± 0.00	0.06 ± 0.01	ns
Oil	14.31 ± 0.32	8.31 ± 0.09	***
Protein (N × 6.25)	87.42 ± 0.48	93.81 ± 0.31	***
Protein (N × 5.7)	79.73 ± 0.44	85.56 ± 0.28	***
Ash	4.77 ± 0.36	6.69 ± 0.70	**
Alkaloids (mg/kg)	607.5 ± 308.6	388.7 ± 110.9	ns
Saponin (g oleanolic acid/100 g)	1.03 ± 0.13	0.86 ± 0.04	ns
**Color parameters**			
L*	77.04 ± 1.18	77.40 ± 0.39	ns
a*	5.10 ± 0.44	4.45 ± 0.10	*
b*	47.66 ± 1.63	42.81 ± 0.37	**

Sig: significance; ns: not significant; *: *p* < 0.05; **: *p* < 0.01; ***: *p* < 0.001.

**Table 7:** Effect of supercritical CO_2_ treatment on amino acid composition (mg/g protein) of lupin protein isolate.

	LPI Type		Sig.
Amino Acids	LPI-Control	LPI-SF	
Aspartic acid	102.07 ± 2.81	108.23 ± 2.02	*
Serine	53.20 ± 0.62	52.74 ± 1.65	ns
Glutamic acid	231.76 ± 4.71	241.46 ± 1.60	**
Glycine	43.12 ± 1.00	43.05 ± 1.27	ns
Arginine	120.33 ± 2.16	112.03 ± 5.31	*
Alanine	31.18 ± 0.34	31.91 ± 0.44	*
Proline	37.25 ± 0.55	36.89 ± 0.81	ns
Cysteine	18.24 ± 0.39	17.81 ± 0.95	ns
Tyrosine	28.83 ± 0.63	28.06 ± 1.80	ns
Non-Essential Aas	665.98 ± 3.12	672.18 ± 1.05	**
Histidine	28.65 ± 0.65	23.98 ± 6.43	ns
Threonine	35.69 ± 0.73	34.10 ± 1.37	ns
Valine	39.29 ± 0.51	39.11 ± 0.83	ns
Methionine	3.38 ± 0.09	4.22 ± 0.13	***
Lysine	55.96 ± 0.98	58.33 ± 1.24	*
Isoleucine	45.32 ± 0.63	45.10 ± 0.97	ns
Leucine	83.35 ± 1.22	82.55 ± 1.67	ns
Phenylalanine	42.38 ± 0.86	40.44 ± 2.44	ns
Essential Aas	334.02 ± 3.12	327.82 ± 1.05	**
E/NE	0.50 ± 0.01	0.49 ± 0.00	*

Sig: significance; ns: not significant; *: *p* < 0.05; **: *p* < 0.01; ***: *p* < 0.001; E/NE: essential/non-essential ratio.

**Table 8:** Effect of supercritical CO_2_ treatment on amino acid chemical score (%) and total indispensable amino acids (mg/g protein) of lupin protein isolate.

		LPI Type		Sig.
	FAO/WHO/UNU [33] (mg/g Protein)	LPI-Control	LPI-SF	
Histidine	10	190.98 ± 4.36	183.31 ± 5.73	ns
Isoleucine	30	151.08 ± 2.11	150.32 ± 3.25	ns
Leucine	59	141.28 ± 2.06	139.91 ± 2.83	ns
Lysine	45	124.35 ± 2.18	129.62 ± 2.75	*
Met + Cys	22	98.30 ± 1.45	100.14 ± 4.70	ns
Methionine	16	21.14 ± 0.59	26.36 ± 0.82	***
Cysteine	6	304.06 ± 6.50	296.91 ± 15.78	ns
Phe + Tyr	38	187.40 ± 3.60	180.26 ± 11.12	ns
Threonine	23	155.17 ± 3.16	148.27 ± 5.94	ns
Valine	39	100.73 ± 1.32	100.29 ± 2.14	ns
Total indispensable amino acids	277	381.09 ± 3.91	373.70 ± 3.12	*

Sig: significance; ns: not significant; *: *p* < 0.05; ***: *p* < 0.001.

## Data Availability

The original contributions presented in this study are included in the article; further inquiries can be directed to the corresponding author.
